# Hemorrhagic Shock Due to a Gastrointestinal Stromal Tumor in a Patient With Neurofibromatosis Type 1: A Case Report

**DOI:** 10.7759/cureus.76028

**Published:** 2024-12-19

**Authors:** Kenneth Garland, Sarthak Parikh, Robert Goodwin

**Affiliations:** 1 Trauma Institute, Saint Francis Health System, Tulsa, USA; 2 Orthopedics, Oklahoma State University Medical Center, Tulsa, USA

**Keywords:** acute care surgery, gastrointestinal stromal tumor (gist), hemorrhagic shock, neurofibromatosis, stromal tumor

## Abstract

Gastrointestinal stromal tumors (GISTs), though rare, are associated with neurofibromatosis (NF) type 1 and may cause significant gastrointestinal bleeding. A 42-year-old male with NF1 presented with severe hematochezia and underwent initial non-contrast CT, which was negative for abnormalities. Subsequent endoscopies and PillCam studies also revealed no clear bleeding source. Due to persistent bleeding and hemodynamic instability, a contrast-enhanced CT was eventually performed, revealing a hyper-enhancing mass in the proximal ileum. Emergent surgical exploration identified a 1.5-cm jejunal GIST, which was resected successfully, stabilizing the patient who was discharged without the need for adjuvant therapy. This case highlights the importance of early contrast-enhanced imaging in NF1 patients presenting with acute bleeding to facilitate timely diagnosis, reduce hospital resource utilization, and avoid unnecessary invasive procedures.

## Introduction

A gastrointestinal stromal tumor (GIST) is an uncommon malignant mesenchymal tumor in the gastrointestinal (GI) tract [1]. According to an analysis of the United States Cancer Statistics from 2001 to 2015, GISTs occur at a rate of 0.70 per 100,000 people per year and have a higher predilection for male (vs. female, 1.27:1) and African American individuals [1]. Although GISTs can affect any part of the GI tract, many cases are localized, and most are present in the stomach followed by the small intestines [1]. Localized disease has the highest five-year overall survival rate at 77% compared with 41% in those with metastatic disease [2].

GISTs arise from activating mutations in the tyrosine kinase receptor KIT or platelet-derived growth factor receptor-α (PDGFR-α) in the interstitial cells of Cajal located within the muscle layer [3]. The tumor is classified as neither benign nor malignant. Nevertheless, approximately 10% to 30% of cases exhibit malignancy in their clinical progression [3]. Studies indicate that both large tumors with elevated mitotic activity and small tumors with low mitotic activity possess the potential for malignant transformation, although the risk is higher in larger, mitotically active tumors [3]. Thus, malignancy risk is stratified based on the pathologic and clinical manifestations.

The clinical presentation of a GIST can include acute melena, hematochezia, hematemesis, subsequent anemia, weakness, abdominal pain, distension, and mass effect. Many patients may also be asymptomatic [4]. Therefore, GISTs may be found incidentally on a diagnostic examination [3]. When presenting acutely, GISTs often present with upper GI bleeding or gastric ulcer-like symptoms [4]. The bleeding can vary in severity. Therefore, it is important that providers keep GISTs high on the differential diagnostic list for patients with acute, life-threatening presentations [4].

Diagnosis of GISTs depends on clinical presentation. In patients with a suspected mass, a computed tomography (CT) scan with oral and IV contrast may illustrate an enhancing solid mass with smooth borders. CT can be used in conjunction with upper endoscopy and endoscopic ultrasound (EUS) to further characterize the tumor and distinguish intramural from extramural lesions. By contrast, colonoscopy can be used to identify GISTs originating in the colon, rectum, or anus. Finally, biopsy can be used for histopathologic examination and classification of spindle cell types, epithelioid types, or mixed types, with spindle cell types accounting for 70% of all GISTs [5]. However, superficial biopsies are often not useful, and techniques such as EUS-guided biopsy or unroofing with subsequent biopsy are typically more effective in obtaining diagnostic tissue samples.

Treatment of non-metastatic GISTs includes endoscopic resection especially if tumor is in favorable location such as the stomach, although recurrence or metastasis occurs in 50% of patients with a median recurrence time of two years [2,3]. Metastatic disease can only be treated with tyrosine kinase inhibitors; however, curative treatment is uncommon. Therefore, early diagnosis is essential for a positive prognosis [3].

Neurofibromatosis type 1 (NF1) is a genetic condition caused by a hereditary deletion mutation of the *NF1* gene on chromosome 17q11.2 [6]. In NF1, the deletion leads to a loss of tumor suppressor function and increases a patient’s susceptibility to developing various tumors. GISTs develop in approximately 7% of patients diagnosed with NF1 [7]. Patients with NF1 are 45 times more likely to develop a GIST compared with their wild-type counterparts; moreover, in this population, GISTs occur at a younger age and more commonly affect women [8]. These tumors tend to be multiple and isolated to the small intestines. They are predominantly spindle cells and have a low metastatic potential. Nevertheless, they still carry a risk of metastasis and clinical complications [6]. The purpose of this case report is to illustrate the acute presentation, diagnosis, and management of a patient with a GIST and a past medical history of NF1. Institutional board approval was obtained.

## Case presentation

A 42-year-old incarcerated male with a history of alcohol use disorder, NF1, and chronic non-steroidal anti-inflammatory drug (NSAID) use presented to the emergency department with 10 episodes of bright red blood per rectum, accompanied by weakness and dizziness. He reported similar symptoms over the preceding several days, including nausea and fatigue. A non-contrast CT scan performed at a clinic visit prior to the onset of significant GI bleeding revealed a small hiatal hernia but no acute abnormalities.

On presentation, his vital signs included a blood pressure of 110/69 mm Hg, heart rate of 109 beats per minute, respiratory rate of 22 breaths per minute, oxygen saturation of 100%, and a lactic acid level of 8.8 mmol/L. Laboratory studies revealed elevated liver enzymes and an increased creatinine level of 1.49 mg/dL. Physical examination was notable for conjunctival pallor but otherwise unremarkable. The patient was admitted for further evaluation and management.

Two days after admission, upper endoscopy revealed mild inflammatory changes in the stomach and duodenum, with no evidence of active bleeding or ulceration. Both perianal and rectal examinations were normal. Despite these findings, the patient experienced continued episodes of large-volume hematochezia, requiring multiple transfusions of packed red blood cells. Lower endoscopy, performed within 24 hours and without terminal ileum intubation, showed no evidence of blood or diverticular disease. A subsequent PillCam endoscopy revealed a few red spots and possible non-bleeding arteriovenous malformations in the proximal to mid jejunum, with no evidence of active bleeding or ulcers. A tagged red blood cell nuclear medicine scan was negative for acute bleeding.

**Figure 1:**
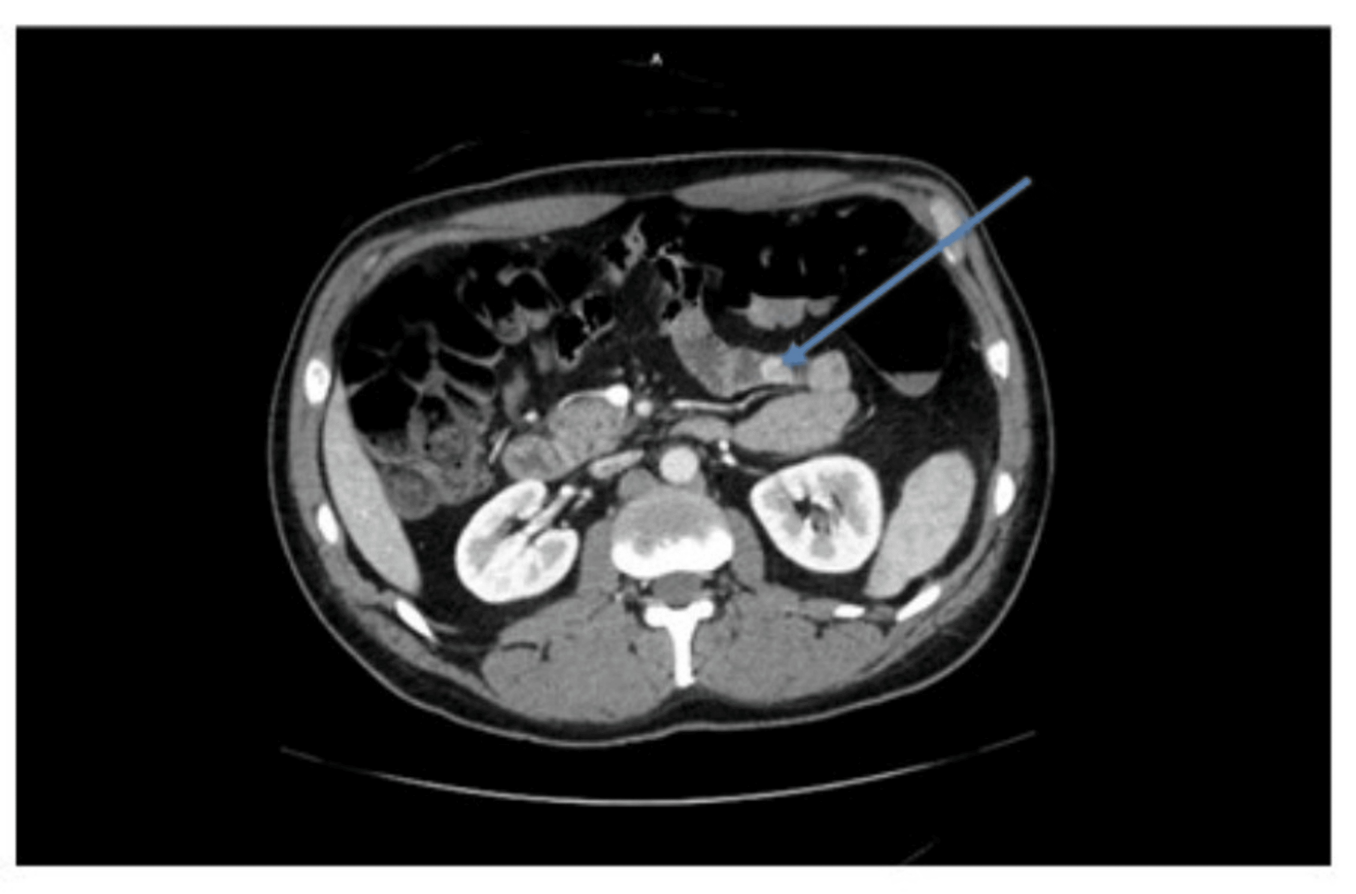
Hyperenhancement of the gastrointestinal stromal tumor within the small bowel on a CT scan with IV contrast

Due to ongoing transfusion requirements, an abdominal-pelvic CT with IV contrast was performed, which demonstrated a hyper-enhancing mass in the proximal ileum without evidence of active bleeding (Figure 1). The patient’s condition deteriorated with persistent hematochezia and hemodynamic instability, consistent with hemorrhagic shock. Acute Care Surgery was consulted, and an emergent laparotomy was performed.

Intraoperatively, a solitary 1.5-cm hyper-enhancing nodule was identified in the mid to distal jejunum. A 5.5-cm segment of jejunum, including the lesion and 5-cm margins, was resected. Pathologic examination revealed a GIST of spindle cell type, measuring 1.5 x 1.3 x 1.2 cm. The tumor had a mitotic rate of 2 mitoses per 5 mm^2^, was G1 low-grade, and exhibited no necrosis or treatment effect. Margins, including a closest proximal margin greater than 1 cm, and one examined lymph node were negative for tumor. Immunohistochemical studies were positive for KIT (CD117) and DOG1 (ANO1). The tumor was staged as pT1, pN0 (AJCC 8th Edition), with no molecular genetic studies performed.

Hematology and Oncology recommended against adjuvant therapy due to the patient’s low risk of recurrence and the absence of additional lesions. The patient stabilized during his hospital stay and was discharged in good condition. At the one-month follow-up, a small left upper lobe pulmonary nodule was noted on imaging but was no longer identifiable at six months. Surveillance CT at 11 months revealed no evidence of metastatic or recurrent disease.

## Discussion

The purpose of this case report is to discuss a case of acute GI hemorrhage caused by a GIST in a patient with NF1 and to offer suggestions for improving management. Despite the patient’s elevated creatinine, a contrast-enhanced CT scan was ordered due to concerns of acute GI bleeding, which outweighed the risks of contrast-induced nephropathy (CIN) [9]. After adequate resuscitation and stabilization of hemodynamics and intravascular volume, CIN can often be corrected [10]. Early resuscitation before performing the contrast CT scan is crucial, as it may lead to earlier detection and treatment of the GIST, ultimately reducing hospital resource utilization, avoiding unnecessary invasive procedures, and shortening the length of stay.

Research published by the Scandinavian Sarcoma Group indicates that our patient did not require adjuvant therapy with imatinib [11]. Joensuu et al. recommend adjuvant treatment with a tyrosine kinase inhibitor for a minimum of three years in patients with completely resected, primary, high-risk GISTs [11]. While specific criteria for identifying high-risk patients are still being established, tumors may be classified as high or low risk based on factors such as tumor size, mitotic rate, location, rupture, and genotype [3,11]. Tumors greater than 10 cm, tumors between 2 and 10 cm with a mitotic index of 5, and tumors in non-gastric locations are considered high-risk and warrant adjuvant therapy. According to the Union for International Cancer Control's "TNM Classification of Malignant Tumors, 8th Edition," our patient has stage-1, low-risk disease, and, as such, adjuvant therapy is not necessary [12].

Neurofibromatosis (NF) is a genetic syndrome characterized by the development of various tumors, with NF1 accounting for 96% of all cases [13]. Literature includes several case reports of GISTs in patients with NF, some of whom also had pheochromocytomas or Hürthle cell tumors [14-16]. The size, mitotic activity, and symptoms of these tumors varied, with some patients being asymptomatic and others presenting with GI hemorrhage [14-16]. No consistent patterns in the presentation of these tumors have been identified, making GIST diagnosis in NF1 patients challenging. We recommend that clinicians maintain a high index of suspicion for GIST in NF1 patients presenting with acute hemorrhagic shock. Additionally, urgent diagnosis with contrast CT, even in the presence of mildly to moderately elevated creatinine levels, may lead to earlier detection of GIST in these patients.

## Conclusions

GISTs, though rare, can present acutely with severe and life-threatening GI bleeding, as demonstrated in this case. Recognizing GIST as a potential cause of acute GI hemorrhage in patients with NF1 is essential for ensuring prompt and effective treatment. Early use of contrast-enhanced CT, despite the risk of CIN, can facilitate timely identification of GIST, reducing the need for extensive resources, invasive procedures, and prolonged hospitalizations. Given the variability in GIST presentation and its potential for serious complications, clinicians managing NF1 patients with unexplained bleeding should maintain a high index of suspicion and prioritize rapid imaging. This proactive approach may improve patient outcomes and streamline care in this unique population.
